# The Hypoxia-Activated Prodrug TH-302: Exploiting Hypoxia in Cancer Therapy

**DOI:** 10.3389/fphar.2021.636892

**Published:** 2021-04-19

**Authors:** Yue Li, Long Zhao, Xiao-Feng Li

**Affiliations:** ^1^ Department of Nuclear Medicine, The Second Clinical Medical College, Jinan University (Shenzhen People’s Hospital), Shenzhen, China; ^2^ The First Affiliated Hospital, Jinan University, Guangzhou, China; ^3^ Department of Nuclear Medicine, The First Affiliated Hospital of Southern University of Science and Technology, Shenzhen, China

**Keywords:** Hypoxia, Hypoxia-activated prodrugs, TH-302, radiotherapy, Chemotherapy

## Abstract

Hypoxia is an important feature of most solid tumors, conferring resistance to radiation and many forms of chemotherapy. However, it is possible to exploit the presence of tumor hypoxia with hypoxia-activated prodrugs (HAPs), agents that in low oxygen conditions undergo bioreduction to yield cytotoxic metabolites. Although many such agents have been developed, we will focus here on TH-302. TH-302 has been extensively studied, and we discuss its mechanism of action, as well as its efficacy in preclinical and clinical studies, with the aim of identifying future research directions.

## Introduction

Hypoxia is an important characteristic of tumors, and generally results in a poor response to radiation and chemotherapy. However, it also presents a therapeutic opportunity, as normal tissue is generally well oxygenated. There have been numerous candidate molecules with enhanced toxicity to hypoxic cells, and they all share a general mechanism: an inert compound is enzymatically reduced to a reactive species, which is easily re-oxidized in the presence of oxygen. Such agents are referred to as hypoxia-activated prodrugs, or HAPs.

The first studies on HAPs were conducted by Alan Sartorelli’s group at Yale, who showed that mitomycin C was preferentially activated under hypoxic conditions, and was thus able to selectively kill hypoxic cells (Lin et al., 1972; Rockwell et al., 1982; Fracasso and Sartorelli, 1986; Pritsos and Sartorelli, 1986). Further HAPs included RSU-1069 and tirapazamine (SR4233) (Laderoute and Rauth, 1986; Whitmore and Gulyas, 1986; Zeman et al., 1986), though neither agent achieved clinical recognition. Recently, a second generation HAP, TH-302 (evofosfamide) has been the subject of extensive preclinical research, much of it supporting the belief that the agent would have a valuable future. However, these hopes were significantly undermined by the failure of phase III clinical trials. Nonetheless, research on TH-302 is still ongoing, and here we will summarize the state of the field.

## Pharmacological Mechanisms

**FIGURE 1:**
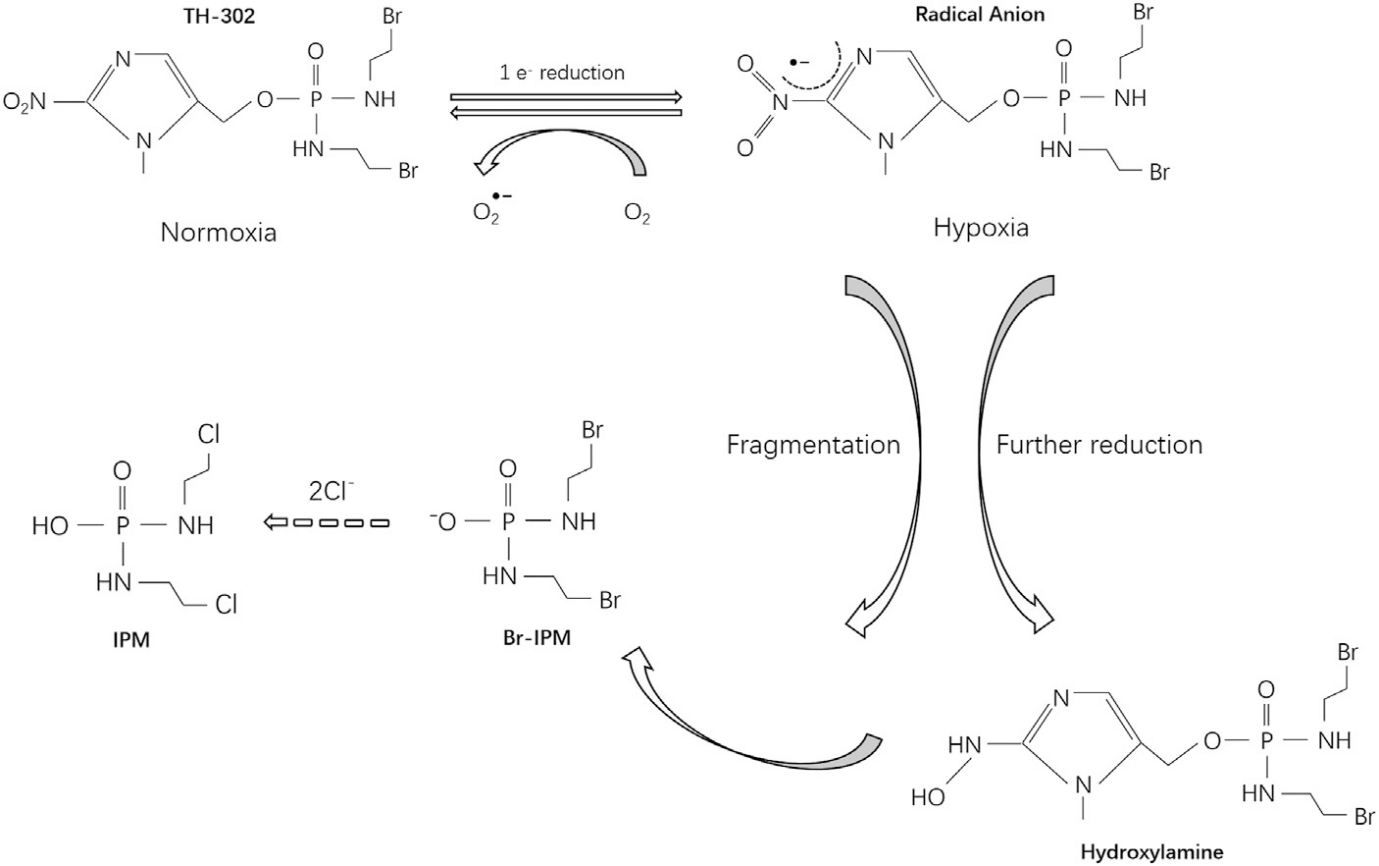
Metabolism of TH-302.

TH-302 was first described in 2008 (Duan et al., 2008). The prodrug consists of a 2-nitroimidazole moiety linked to bromo-iso-phosphoramide mustard (Br -IPM), a DNA cross-linking agent. TH-302 is a substrate for certain cellular reductases that generate a radical anion through 1-electron reduction. Under normoxic conditions, the free radical anions are quickly oxidized back to either the original prodrug or superoxides, and no cytotoxic product is released. However, in the absence of oxygen, the free radical anions are further reduced, leading to the release of Br-IPM or its stable downstream product, isophosphoramide mustard (IPM) (Figure 1). The reductase involved in this selective activation under hypoxia is not yet fully understood. However, Hunter et al. investigated potential modifiers of TH-302 metabolism by RNA sequencing, whole-genome CRISPR knockout, and reductase-focused short hairpin RNA screens, and found that the activation of TH-302 is related to genes involved in mitochondrial electron transfer, DNA damage-response factors and mitochondrial function regulators, such as *SLX4IP*, *C10orf90 (FATS)*, *SLFN11*, *YME1L1* (Hunter et al., 2019).

TH-302 shows obvious biliary excretion and/or gut secretion (Jung et al., 2012), with a short half-life of 12.3 min, a high clearance rate of 2.29 L/h/kg, and its volume of distribution is 0.627 L/kg.

## Preclinical Studies

### *In vitro* Cytotoxicity

In a panel of 32 human cancer lines, Meng et al. found that all cells displayed enhanced sensitivity to TH-302 under severely hypoxic conditions (∼0.1% O_2_). Consistent with enhanced cell killing, TH-302/hypoxia also induced γH2AX phosphorylation, DNA cross-linking and cell cycle arrest. Additional studies with repair deficient CHO cells found that loss of homologous repair increased drug sensitivity; non-homologous end-joining, base and nucleotide excision played no role in processing the DNA/IPM lesions (Meng et al., 2012). Also consistent with a DNA damage response, TH-302/hypoxia can down-regulate levels of the three D cyclins, as well as CDK4/6, p21 (cip-1) p27 (kip-1), and phosphorylated Rb, and up-regulate the expression of caspases-3,8 and 9, and poly ADP-ribose polymerase to induce both G0/1 cell cycle arrest and trigger apoptosis in multiple myeloma (Hu et al., 2010). TH-302 decreased proliferation and HIF-1α expression in acute myeloid leukemia (AML) and nasopharyngeal carcinoma (NPC) cells and induced cell-cycle arrest, and enhanced double-stranded DNA breaks (Portwood et al., 2013; Huang et al., 2018). TH-302 was selectively toxic to hypoxic (1% O_2_) osteosarcoma cells while normal osteoblasts were protected (Liapis et al., 2015). The combination of TH-302 with cisplatin (DDP) had a synergistic effect on cytotoxicity in nasopharyngeal cancer cell lines (Huang et al., 2018). Under hypoxic conditions (1% O_2_), TH-302 significantly inhibited the survival of melanoma cells in two/three-dimensional (2D/3D) culture, and the combination with sunitinib further enhanced the effect (Liu et al., 2017).

In 3D tumor spheroids and multi-cellular layer models, TH-302 was more effective in tumor spheroids compared with monolayer cells, indicating that TH-302 had a significant “bystander effect” (Meng et al., 2012; Voissiere et al., 2017). Ham et al. showed that in a 3D breast cancer cell (MDA-MB-157) model, the combination treatment with doxorubicin and TH-302 could significantly reduce drug resistance (Ham et al., 2016).

### Response of Experimental Tumors

#### Monotherapy

Single agent TH-302 has shown efficacy against multiple human xenografts, including hepatoma, multiple myeloma (MM), neuroblastoma, rhabdomyosarcoma, osteolytic breast cancer, non-small cell lung cancer (NSCLC), head and neck squamous cell carcinoma (HNSCC), and acute myeloid leukemia (Hu et al., 2010; Li et al., 2010; Portwood et al., 2013; Liapis et al., 2016; Sun et al., 2016; Zhang et al., 2016a; Harms et al., 2019). Using two high-grade glioma models (C6 glioblastoma and 9 L glioma) with different levels of hypoxia, Stokes et al. showed that the more hypoxic, less perfused C6 tumor model was more sensitive to TH-302 (Stokes et al., 2016).

A study by Sun et al. further supported the “bystander effect” of TH-302 in animal models. They found that the DNA damage induced by TH-302 initially only appeared in hypoxic regions, but subsequently spread to the entire tumor (Sun et al., 2012). However, the bystander hypothesis was questioned by Hong et al. who found that the toxic metabolites Br-IPM and IPM were unable to pass across cell membranes. They proposed that any effect on oxygenated tumor cells was due to high concentrations of pro-drug leading to some residual Br-IPM formation even in the presence of oxygen. (Hong et al., 2018; Hong et al., 2019).

Nytko et al. demonstrated that the efficacy of TH-302 is highly dependent on tumor type, largely due to levels of cytochrome P450 oxidoreductase activity (POR) (Nytko et al., 2017). Through the study of 22 cases of papillomavirus-negative head and neck squamous cell carcinoma (HPV-negative HNSCC), Jamieson et al. confirmed that for hypoxic HPV-negative HNSCC cells, TH-302 exhibited stronger potency and selectivity than the previous generation HAP (PR- 104 A or SN30000), and the responsiveness was dependent on the sensitivity to DNA cross-linking and the activation rate of the prodrug. They also revealed the correlation between TH-302 sensitivity and proliferative rate/proliferation metagene (Jamieson et al., 2018). Recent evidence suggests that TH-302 can not only kill hypoxic pancreatic cancer cells, but also has the ability to improve the oxygenation status of residual tumor cells, so it can be used to enhance the effect of radiotherapy and chemotherapy (Kishimoto et al., 2020) (Table 1).

**TABLE 1:** Pre-clinical studies of TH-302.

Ref	Tumor type (Cell lines/tumor models)	Combined therapy					
		Radioherapy	Chemotherapy	Anti-angiogenic agents	Molecular targeted agents	Immunoherapy	Other therapy
Li et al. (2010)	Hepatoma (H22)	—	—	—	—	—	—
Hu et al. (2010)	Multiple myeloma (5T33 MM model)	—	—	—	—	—	—
Sun et al. (2012)	11 xenograft models	—	—	—	—	—	—
Meng et al. (2012)	Chinese hamster ovary cell, H460, H116	—	—	—	—	—	—
Liu et al. (2012)	11 human xenograft models	—	Docetaxel, cisplatin, pemetrexed, irinotecan, doxorubicin, gemcitabine, temozolomide	—	—	—	—
Portwood et al. (2013)	Acute myeloid leukemia (HEL, HL60)	—	—	—	—	—	—
Saggar and Tannock (2014)	Breast cancer (MCF-7)/prostate caner (PC-3)	—	Docetaxel Doxorubicin	—	—	—	—
Takakusagi et al. (2014)	Squamous cell carcinoma (SCCVII)/Adenocarcinoma (HT29)	—	Pyruvate	—	—	—	—
Bailey et al. (2014)	PDAC (MiaPaCa-2, SU.86.86)	—	Hydralazine	—	—	—	—
Wojtkowiak et al. (2015)	PDAC (Hs766t, MiaPaCa-2, SU.86.86)	—	Pyruvate	—	—	—	—
Liapis et al. (2015)	Osteosarcoma	—	Docetaxel	—	—	—	—
Saggar and Tannock (2015)	Breast cancer (MCF-7)/prostate caner (PC-3)		docetaxel, doxorubicin	—	—	—	—
Sun et al. (2015a)	Renal cell carcinoma (786-O, Caki-1)	—	Everolimus/Temsirolimus (mTOR inhibitor)	—	—	—	—
Yoon et al. (2015)	Sarcoma	RT	—	DC101(VEGF-A inhibitor)			
Peeters et al. (2015)	NSCLC and rhabdomyosarcoma	RT	—	—	—	—	—
Sun et al. (2015b)	PDAC (Hs766t, MIA PaCa-2, PANC-1, and BxPC-3)	—	gemcitabine,nab-paclitaxel	—	—	—	—
Liapis et al. (2016)	Osteolytic breast cancer (MDA- B- 31- XSA)	—	Paclitaxel	—	—	—	—
Sun et al. (2016)	NSCLC (H460)	—	Docetaxel	Sunitinib	—	—	—
Benito et al. (2016)	Leukemia (KBM-5, KG-1, OCI-AML3, MOLM-13, REH, Nalm-6)	—	—	—	Sorafenib	—	—
Lohse et al. (2016)	Pancreatic cancer (PDX model)	IR	—	—	—	—	—
Zhang et al. (2016a)	Neuroblastoma/rhabdomyosarcoma	—	Topotecan	—	—	—	—
Yoon et al. (2016)	Undifferentiated pleomorphic sarcoma (KP mice model)	—	Low dose doxorubicin (HIF-1α inhibitor)	DC101(VEGF-A inhibitor)	—	—	—
Lindsay et al. (2016)	EGFR-mutant NSCLC	—	—	—	Erlotinib	—	—
Stokes et al. (2016)	Glioma (C6 glioblastoma/9 L gliosarcoma)	—	—	—	—	—	—
Ham et al. (2016)	Breast cancer (MDA-mb-157)	—	Doxorubicin	—	—	—	—
Duran et al. (2017)	Hepatocellular carcinoma (VX2)	—	—	—	—	—	cTACE (doxorubicin)
Nytko et al. (2017)	Lung adenocarcinoma (A549)/HNSCC (UT-scc-14)	Fractionated IR	—	—	—	—	—
Voissiere et al. (2017)	Chondrosarcoma (HEMC-SS)	—	—	—	—	—	—
Liapis et al. (2017)	Osteosarcoma (BTK-143, K-OS)	—	Dulanermin/drozitumab	—	—	—	—
Hajj et al. (2017)	Pancreatic cancer (AsPC1)	RT	—	—	—	—	—
Liu et al. (2017)	Melanoma (WM35, WM793, 1205LU)	—	—	Sunitinib	—	—	—
Takakusagi et al. (2018)	Squamous cell carcinoma (SCCVII)/Adenocarcinoma (HT29)	IR	—	—	—	—	—
Huang et al. (2018)	NPC (CNE-2, HONE-1, HNE-1)	—	Cisplatin (DDP)	—	—	—	—
Haynes et al. (2018)	Colorectal cancer (PDX model)	RT	5-Fu	—	—	—	—
Conway et al. (2018)	PDAC (KPC primary PDAC cells)	—	AZD2014	—	—	—	—
Jamieson et al. (2018)	HNSCC (SCC-4, SCC-7, SCC-9, FaDu, UT-SCC and PDX model)	—	—	—	—	CTLA-4 blockade	—
Kumar et al. (2018)	Neuroblastoma (SK-N-BE (2))	—	—	Sunitinib	—	—	—
Hong et al. (2018)	Colon carcinoma (HCT116)	—	—	—	—	—	—
Jayaprakash et al. (2018)	Prostate cancer (TRAMP-C2)	—	—	—	—	αCTLA-4/αpd-1	—
Hong et al. (2019)	NSCLC (H460)	—	—	—	—	—	—
Harms et al. (2019)	HNSCC (PDX model)	—	—	—	—	—	—
Spiegelberg et al. (2019)	Esophageal carcinomas (OE19/OE21)	RT	—	—	—	—	—

#### Combination of TH-302 With Conventional Chemotherapy

TH-302 has been shown to enhance the anti-tumor effect of many conventional chemotherapy drugs, such as docetaxel, cisplatin, pemetrexed, irinotecan, doxorubicin, gemcitabine, temozolomide, and topotecan (Liu et al., 2012; Saggar and Tannock, 2014; Liapis et al., 2015; Sun et al., 2015b; Zhang et al., 2016b; Liapis et al., 2016; Huang et al., 2018). Saggar and Tannock demonstrated that TH-302 could inhibit tumor reoxygenation and as well as the proliferation of hypoxic tumor cells that survived chemotherapy (Saggar and Tannock, 2015).

For the treatment of osteosarcoma, TH-302 combined with proapoptotic receptor agonists (dulanermin or drozitumab) or doxorubicin could effectively reduce the tumor burden of bone as well as pulmonary metastases and could prevent bone destruction caused by osteosarcoma (Liapis et al., 2015; Liapis et al.,2017).

As cancer-initiating cells (C-ICs) are associated with hypoxic niches, Haynes et al. investigated and proposed that conventional treatments such as fluorouracil with or without radiotherapy, would enhance tumor hypoxia and thus expand the C-IC population, which could be counteracted by TH-302 treatment (Haynes et al., 2018). The PI3K pathway is involved in cell adaptation to hypoxia, via Akt mitochondrial translocation (Chae et al., 2016). However, in pancreatic ductal adenocarcinoma (PDAC) cells, resistance to the PI3K pathway inhibitor was associated with tumor hypoxia. Conway et al. combined TH-302 and AZD2014 for the treatment of tumor-bearing mice. The results showed that single use of AZD2014 improved survival and had additional anti-invasive effects, while TH-302 as a single agent exhibited higher efficacy under hypoxic conditions. As expected, the combination of TH-302 and AZD2014 enhanced the potency of each drug, ultimately leading to an overall improvement in anti-tumor effects (Conway et al., 2018).

#### Combination of TH-302 With Radiotherapy

Since hypoxic cells are known to be extremely radioresistant, there is a powerful rationale for combing radiation and TH-302. Several investigators have demonstrated increased tumor growth delay and decreased hypoxic fraction in a variety of tumor types (NSCLC, rhabdomyosarcoma, squamous cell carcinoma, colorectal adenocarcinoma, pancreatic cancer) when using this combination (Peeters et al., 2015; Hajj et al., 2017; Nytko et al., 2017; Takakusagi et al., 2018). Lohse et al. studied 11 pancreatic cancer PDX models and found that the combination of TH-302 and ionizing radiation (IR) could significantly delay tumor growth, reduce tumor volume, and reduce the frequency of tumor initiating cells (TIC), especially in the more rapidly growing/hypoxic models (Lohse et al., 2016). Spiegelberg et al. confirmed that TH-302 could increase the sensitivity of esophageal carcinoma to radiotherapy, without any additional toxicity to the gastrointestinal tract (mucosal damage) and lung (fibrosis) (Spiegelberg et al., 2019b).

#### Combination of TH-302 With Tissue Oxygen Modulators or Anti-Angiogenic Therapy

Any treatment that increases tumor hypoxia might be expected to enhance the response to TH-302. For example, pretreatment with pyruvate has been shown to increase TH-302 sensitivity, through increased mitochondrial oxygen consumption and concomitant transient tumor hypoxia (Takakusagi et al., 2014; Wojtkowiak et al., 2015). Hydralazine (a vasodilator) that is known to profoundly exacerbate hypoxia in murine tumors, enhanced the efficacy of TH-302 (Bailey et al., 2014).

However, the most obvious candidates for such an approach are anti-angiogenics. In two renal cell carcinoma models, the mTOR inhibitors everolimus and temsirolimus both reduced vessel density, with resultant increase in hypoxia and TH-302 response (Sun et al., 2015a). Yoon et al. combined TH-302 with the VEGF-A inhibitor DC101, a HIF-1α inhibitor (low-dose doxorubicin) and radiotherapy for the treatment of mouse models of sarcoma. The results showed that this multi-modal therapy could effectively block sarcoma growth. The mechanism involved the increase of DNA damage and apoptosis in endothelial cells, the reduction of HIF-1α activity, and the inhibition of cancer stem cell-like cells (Yoon et al., 2015; Yoon et al., 2016). Experiments conducted by Kumar et al. using a subcutaneous xenograft model of neuroblastoma showed that the combined use of TH-302 and sunitinib (an anti-angiogenic multikinase inhibitor) resulted in greater tumor growth delay, increased apoptosis and tumor hypoxia. They also found that the combination therapy significantly reduced the burden of liver metastases (Kumar et al., 2018). With genetically engineered melanoma mouse models, Liu et al. showed that while sunitinib alone would lead to greater hypoxia without tumor suppression, TH-302 in combination with sunitinib could significantly reduce tumor volume and prolong survival (Liu et al., 2017).

#### Combination of TH-302 With Molecular Targeted Therapy

Benito et al. found that the combination of TH-302 and sorafenib resulted in greater anti-leukemia efficacy than either alone (Benito et al., 2016). Lindsay et al. established a stochastic mathematical model, parameterized experimental and clinical data, and concluded that the combination therapy of TH-302 and erlotinib was better than single-agent therapy of either in EGFR-mutant NSCLC, which was mainly reflected in delayed drug resistance (Lindsay et al., 2016).

#### Combination of TH-302 With Immunotherapy

A new and promising way to exploit TH-302 may be in combination with immunotherapy. Jayaprakash et al. demonstrated that the hypoxic regions in the prostate cancer models lacked T cell infiltration, potentially creating zones of immunotherapy resistance. To overcome this, they combined TH-302 with a maximal checkpoint blockade directed against both CTLA-4 and PD-1, dramatically enhancing the effect of the immunotherapy treatment (Jayaprakash et al., 2018). Likewise, Jamieson et al. also found that the combined therapy of TH-302 and CTLA-4 blockade can further improve the survival rate of the HNSCC model compared with single use either alone (Jamieson et al., 2018).

#### Combination of Th-302 With Other Therapies

For the treatment of hepatocellular carcinoma, Duran et al. used hepatic hypoxia activated intra-arterial therapy (HAIAT) and found that the addition of TH-302 to conventional Trans Arterial ChemoEmbolization (cTACE) achieved promising anti-cancer effects, which mainly manifested as reduced tumor burden, decreased tumor growth rate and increased necrotic fraction (Duran et al., 2017).

## Clinical Trials

TH-302 entered clinical trials in 2007 and results were first reported in 2011 (Table 2). Weiss et al. enrolled 57 patients with advanced solid tumors who were treated with TH-302 monotherapy (dose and scheme: TH-302 was administered i. v. over 30–60 min. Arm A: 7.5–670 mg/m^2^, 3 times weekly dosing followed by 1 week off; Arm B: 670–940 mg/m^2^, every 3 weeks dosing). They reported skin and/or mucosal toxicity with a maximum tolerated dose (MTD) of 670 mg/m^2^. They observed two partial responses and 27 cases of stable disease. Additionally, TH-302 helped to resolve Cullen’s sign in patients with metastatic melanoma (Weiss et al., 2011a, Weiss et al., 2011b). Riedel et al. conducted a phase one clinical trial on 30 patients with advanced solid tumors. Their results revealed the potential therapeutic value of co-targeting tumor angiogenesis and hypoxia (dose and scheme: pazopanib, orally dosed at 800 mg daily on days 1–28; TH-302, administered i. v. on days 1, 8, and 15 of a 28 days cycle at doses of 340 or 480 mg/m^2^) (Riedel et al., 2017). Conroy et al. reported the efficacy of TH-302 as a monotherapy on two patients with advanced ovarian serous carcinoma with BRCA1 mutations. Both individuals responded well (dosed at either 300 mg/m^2^ (9 cycles, 15 months) or 340 mg/m^2^ (6 cycles, 3 months)) showing partial response or stable disease (Conroy et al., 2017). A phase one surgical study of TH-302 (dose range 240–670 mg/m^2^, every 2 weeks) combined with bevacizumab (dose: 10 mg/kg) in the treatment of bevacizumab-refractory glioblastoma found that the therapy was well-tolerated at 670 mg/m^2^, with an overall response rate of 17.4% and a disease control rate of 60.9% (Brenner et al., 2018). The phase 1/2 study of TH-302 (NCT01522872) conducted by Laubach et al. showed that for relapsed/refractory myeloma, TH-302 alone or in combination with bortezomib was well tolerated and could prolong survival (dose and scheme: Arm A: 340 mg/m^2^ dose of TH-302 was administered i. v. over 30–60 min with a fixed oral 40 mg dose of dexamethasone on days 1, 4, 8 and 11 of a 21 days cycle; Arm B: 340 mg/m^2^ dose of TH-302 was administered i. v. over 30–60 min with a fixed oral 40 mg dose of dexamethasone and a fixed i. v. or s. c. administration of 1.3 mg/m^2^ dose of bortezomib on days 1, 4, 8, and 11 of a 21 days cycle) (Laubach et al., 2019). The anti-tumor effect of TH-302 (300 mg/m^2^ administered i. v. on days 1 and 8 of each 21 days cycle, 6 cycles) combined with doxorubicin (75 mg/m^2^ administered i. v. on day 1 of each 21 days cycle, 6 cycles) in the treatment of advanced soft tissue sarcoma (STS) has also been tested in phase two clinical trials, and complete and partial responses have been observed (Chawla et al., 2014). Borad et al. evaluated the therapeutic effect of TH-302 combined with gemcitabine on pancreatic cancer. Prolonged progression-free survival (PFS) and CA19–9 response were observed (dose and scheme: 240 or 340 mg/m^2^ TH-302 administered i. v. over 30–60 min followed 2 h later by a 30 min i. v. infusion of gemcitabine 1,000 mg/m^2^ on days 1, 8, and 15 of each 28 days cycle). Skin and mucosal toxicity and bone marrow suppression are the most common toxicities (Borad et al., 2015). Another phase two study enrolled five HNSCC patients receiving TH-302 monotherapy (480 mg/m^2^ qw × 3 each month). Two of them achieved partial response, and the other three had stable disease (Jamieson et al., 2018).

**TABLE 2:** Clinical trials of TH-302.

Ref	Tumor type	Clinical trial	Number of patients	Combined therapy
Weiss et al. (2011a)	Solid tumors	Phase 1	57	—
Ganjoo et al. (2011)	Soft tissue sarcoma	Phase 1	16	Doxorubicin (chemotherapy)
Weiss et al. (2011b)	Melanoma	Phase 1	1	—
Chawla et al. (2014)	Soft tissue sarcoma	Phase 2	91	Doxorubicin (chemotherapy)
Borad et al. (2015)	Pancreatic cancer	Phase 2	214	Gemcitabine (chemotherapy)
Van Cutsem et al. (2016)	Pancreatic cancer	Phase 3	660	Gemcitabine (chemotherapy)
Badar et al. (2016)	Leukemia	Phase 1	49	—
Riedel et al. (2017)	Advanced solid tumors	Phase 1	30	Pazopanib (anti-angiogenic agents)
Conroy et al. (2017)	Ovarian serous carcinoma	Case report	2	—
Tap et al. (2017)	Soft-tissue sarcoma	Phase 3	640	Doxorubicin (chemotherapy)
Brenner et al. (2018)	Glioblastoma	Phase 1	28	Bevacizumab (anti-angiogenic agents)
Jamieson et al. (2018)	HNSCC	Phase 2	5	—
Laubach et al. (2019)	Multiple myeloma	Phase 1/2	59	Bortezomib (chemotherapy)

TH-302 was successfully applied in the clinic but the outcomes were not sufficient to receive approval from requlatory authorities. Badar et al. revealed that TH-302 exhibited limited activity in leukemia patients (doses ranging between 120 and 550 mg/m^2^) (Badar et al., 2016). In the phase three multicenter clinical trial (TH CR-406/SARC021), 640 patients with soft tissue sarcoma were enrolled. The results showed that the combination of TH-302 (300 mg/m^2^ administered i. v. for 30–60 min on days 1 and 8 of every 21 days cycle, 6 cycles) and doxorubicin (75 mg/m^2^ administered on day 1 of every 21 days cycle, six cycles) failed to improve overall survival compared with doxorubicin alone (Tap et al., 2017). But it should be noted that the historical survival benefit of doxorubicin monotherapy shows a trend for improvement over time, perhaps due to superior clinical management of associated toxicities. The initial phase two combination study (Dox + TH-302) was a single arm study that utilized historical doxorubicin single agent survival results (12–13 months) as reference. Ultimately this proved to be an invalid comparison. In addition, antagonistic effects between drugs (Anderson et al., 2017) and changes in drug formulations (Higgins et al., 2018) should also be considered as potential causes. TH-302 plus gemcitabine in the treatment of patients with pancreatic ductal adenocarcinoma (PDAC) also missed the end point of another phase three clinical trial (dose and scheme: TH-302 340 mg/m^2^ and gemcitabine 1,000 mg/m^2^ administered i. v. on days 1, 8, and 15 of a 28 days cycle) (NCT01746979) (Van Cutsem et al., 2016). In this case, lack of patient screening based on tumor hypoxia may have been the most important cause of the trial’s failure (Domenyuk et al., 2018; Spiegelberg et al., 2019a). In contrast to the prevalent belief that all PDAC are severely hypoxic, evidence showed that the levels of hypoxia observed in PDAC were highly heterogeneous (range from 0 to 26%) and were similar to those reported in other tumor types (Dhani et al., 2015). Patients with a low tumor hypoxic fraction are not expected to benefit from TH-302 treatment, and a more efficient approach to the clinical application of TH-302 may be to determine the tumor hypoxic status of tumor prior to patient selection.

## Discussion and Directions for Future Applications

Hypoxia is an important feature of solid tumors and may also be an effective new target for tumor therapy. We are trying to put forward new suggestions on the clinical application of TH-302 or other HAPs. Hypoxia is not only a characteristic of macroscopic tumors. In 2007, our group reported that peritoneal disseminated micro-metastases (less than 1 mm in diameter) are severely hypoxic and poorly proliferative (Li et al., 2007; Li and O’Donoghue, 2008; Li et al., 2010b; Li et al., 2010a; Huang et al., 2013). Further, our data indicated that tumor cells in these hypoxic micro-metastases could survive for several weeks (data to be published). In view of this special state of early micro-metastases of tumors, TH-302 may have the potential to prevent them from developing into macroscopic tumors, thereby reducing the recurrence and metastasis rate of tumors. In this area, TH-302 may be superior to traditional radiotherapy and chemotherapy. Our group is conducting further research.
